# Family Metacognitive Training (MCT-F): Adapting MCT to Mothers with Psychosis and Their Adolescent Children

**DOI:** 10.3390/bs14020097

**Published:** 2024-01-27

**Authors:** Victoria Espinosa, Paula Arin-González, Alba Jiménez-Lafuente, Nerea Pardo, Raquel López-Carrilero, Irene Birulés, Ana Barajas, Trinidad Pélaez, Luciana Díaz-Cutraro, Marina Verdaguer-Rodríguez, Alfonso Gutiérrez-Zotes, Carolina Palma-Sevillano, Paloma Varela-Casals, Miriam Salas-Sender, Ana Aznar, Rosa Ayesa-Arriola, Esther Pousa, Manuel Canal-Rivero, Nathalia Garrido-Torres, Clara Montserrat, Laura Muñoz-Lorenzo, Josep Maria Crosas, Susana Ochoa

**Affiliations:** 1 Parc Sanitari Sant Joan de Déu, 08830 Sant Boi de Llobregat, Spain; victoria.espinosa@sjd.es (V.E.); paula.arin@sjd.es (P.A.-G.); alba.jimenez@sjd.es (A.J.-L.); nerea.pardo@sjd.es (N.P.); raquel.lopezc@sjd.es (R.L.-C.); irene.birules@sjd.es (I.B.); mtrinidad.pelaez@sjd.es (T.P.); luciana.diaz@sjd.es (L.D.-C.); marina.verdaguer@sjd.es (M.V.-R.); 2 Fundació Sant Joan de Déu, 08950 Esplugues de Llobregat, Spain; 3 Etiopatogènia i Tractament dels Trastorns Mentals Greus (MERITT), Institut de Recerca Sant Joan de Déu, 08950 Esplugues de Llobregat, Spain; 4 Centro de Investigación Biomédica en Red de Salud Mental, Instituto de Salud Carlos III, 28029 Madrid, Spain; gutierreza@peremata.com (A.G.-Z.); rosayesa@gmail.com (R.A.-A.); epousa@santpau.cat (E.P.); 5 Department of Social and Quantitative Psychology, Faculty of Psychology, University of Barcelona, 08035 Barcelona, Spain; 6 Departament de Psicologia, Facultat de Psicologia Clínica I de la Salut. Serra Húnter Programme, Universitat Autònoma de Barcelona, 08193 Cerdanyola del Vallès, Spain; ana.barajas@uab.cat; 7 Psychology Department, FPCEE Blanquerna, Universitat Ramon Llull, 08022 Barcelona, Spain; carolinaps@blanquerna.url.edu; 8 Department of Clinical and Health Psychology, Universitat Autònoma de Barcelona, 08193 Cerdanyola del Vallès, Spain; 9 Hospital Universitari Institut Pere Mata, Institut d’Investigació Sanitària Pere Virgili-CERCA, Universitat Rovira i Virgili, 43206 Reus, Spain; 10 Hospital de Mataró, Consorci Sanitari del Maresme, 08301 Mataró, Spain; pvarela@csdm.cat; 11 Fundació Vidal i Barraquer, 08022 Barcelona, Spain; miriam.salas.sender@gmail.com; 12 Centre d’Higiene Mental Les Corts, 08035 Barcelona, Spain; ana.aznar@chmcorts.com; 13 Department of Psychiatry, Marqués de Valdecilla University Hospital, IDIVAL. School of Medicine, University of Cantabria, 39008 Santander, Spain; 14 Department of Psychiatry, Hospital de la Santa Creu i Sant Pau, Institut d’Investigació Biomèdica-Sant Pau (IIB-Sant Pau), 08041 Barcelona, Spain; 15 Virgen del Rocío University Hospital, Institute of Biomedicine of Seville (IBiS), University of Seville, First-Episode Psychosis Research Network of Andalusia (Red PEPSur), 41013 Sevilla, Spain; mcanalrivero@gmail.com (M.C.-R.); nagatorr@gmail.com (N.G.-T.); 16 Hospital del Mar Medical Research Institute (IMIM) of Barcelona, Autonomous University of Barcelona, 08003 Barcelona, Spain; cmontserrat@parcdesalutmar.cat; 17 Departamento de Psiquiatría, IIS-Fundación Jiménez Díaz, 28040 Madrid, Spain; lauramlorenzo@hotmail.com; 18 Department of Mental Health, Hospital Universitari Parc Taulí, Institut d’Investigació i Innovació Parc Taulí I3PT, Universitat Autònoma de Barcelona, 08208 Sabadell, Spain; jcrosas@tauli.cat

**Keywords:** mothers with psychosis, adolescents’ mental health, metacognitive training, ADAPT-ITT framework, family intervention

## Abstract

Over half of women with psychosis are mothers. Research suggests that mothers with psychosis face unique challenges affecting both their mental health prognosis and their relationship with their children. Moreover, those children have a higher risk of developing a mental disorder. Notwithstanding, interventions specifically tailored to these families remain largely uncovered. Metacognitive Training (MCT) has demonstrated its efficacy in improving cognitive insight, symptom management, and social cognition in people with psychosis. However, there is no evidence of the efficacy of MCT in a family setting (MCT-F). This study describes the first adaptation of MCT for mothers with psychosis and their adolescent children in an online group setting. The phases (assessment, decision, adaptation, production, topical experts’ integration) of the ADAPT-ITT model were systematically applied through a participatory approach (*n* = 22), including a first-person perspective and involving qualitative (e.g., topical expert literature review and consensus groups, interviews, thematic analyses) and quantitative methods. While MCT’s core components were retained, participants guided adaptations both in content and delivery. The findings suggest the importance of community engagement and sharing decision-making processes to demonstrate the acceptability and feasibility of the adapted intervention. Employing a structured approach such as the ADAPT-ITT model ensures readiness of the new training for efficacy trials.

## 1. Introduction 

Psychotic disorders affect roughly 2% of the population, and their chronic course may cause severe levels of disability. Thus, psychosis often leads to a considerable burden for caregivers and health systems [1]. Importantly, a total of 50% of women with schizophrenia and 60% of women with psychosis are mothers [2,3,4]. The proportions in this clinical population are similar to those in women without a diagnosis of a mental disorder [5]. In comparison to their male counterparts, mothers with psychosis were more likely to be a single parent, perform most parenting tasks, or report having parenting-related stress [6].

Research on motherhood in psychosis is not only scarce [3] but also focuses on mothers experiencing postpartum psychosis [7,8,9]. However, mothers with psychosis encounter unique difficulties associated with their mental health condition. Some of them are related to their illness’ severity, problems in reasoning biases, attributional errors, the presence of social stressors and self-stigma associated with the illness, as well as a lack of protective factors to deal with these issues [8], in which social cognitive impairments play a prominent role [10]. 

Such deficits affect the mothers’ understanding of their children’s mental states and their ability to engage in positive and nurturing interactions with them [11]. The symptoms of psychosis, such as delusions or hallucinations, leave parents unable to focus on their child’s needs [9] and can also incite confusion and fear in the children [12]. Furthermore, the stigma associated with mental illness may affect the child’s perception of their mother and hinder their ability to seek support and understanding, experiencing their mother’s disorder as a family secret [13]. Consequently, parents with serious mental illness, including psychosis, are at higher risk of custody loss compared to parents without mental health problems [14]. These custody issues can further exacerbate the challenges faced by mothers with psychosis and their children.

Having a family member with psychosis is a widely replicated risk factor for the development of mental health problems [15,16]. It is estimated that more than 70% of children living with parents with mental health problems will experience mental health difficulties themselves [17]. These challenges can arise due to a combination of genetic factors, such as the heritability of X-linked psychosis [18], and environmental factors, including problems in family dynamics [19]. The symptoms observed in these children can range from emotional and behavioral problems to cognitive impairments and social difficulties [16], which become more evident in adolescence. While interventions targeting high-risk adolescents have shown some effectiveness in preventing the transition to psychosis, these interventions are typically delivered after the onset of subclinical symptoms or a decline in functioning [20].

Despite the evidence demonstrating the issues that adolescent children of mothers with psychosis face, limited research exists on developing and evaluating interventions specifically tailored to mothers with psychosis and their adolescent children [21]. Firstly, most interventions have been designed for parents with severe mental illness in general [22]. Only one intervention, the Kidstime project by Wolpert et al. [23], included a high percentage of parents with psychosis in their sample. Secondly, few studies have specifically targeted mothers [24] or the adolescent period [25]. During adolescence, social cognition, emotional recognition, theory of mind, attributional style, and social perception- become instrumental in social relationships and in the development of the social brain [26,27]. Thirdly, although results suggest that most family interventions have good levels of acceptability in parents, participants have reported preferring interventions where the illness is not the central focus, which results in a lack of emphasis on the parents and their needs, such as the desire for additional support for their children [11]. Finally, although interventions focusing solely on psychoeducation about parental mental illness or parenting skills have been shown to be insufficient in improving cognitive biases and social cognition [23,28], many interventions for mental disorders are still addressed mainly to increase psychoeducation [21].

Metacognitive Training (MCT) [29] has emerged as a promising psychological intervention for individuals with psychosis. It focuses on improving cognitive insight, symptom management, and social cognition [30]. However, MCT has primarily been delivered to people with psychosis, or even in the early stages of the illness [31] but has not yet been adopted as a family intervention. Additionally, MCT has shown better results in females than males with psychosis [32]. Importantly, it has been suggested that online therapy may often be preferred by female clients, due to their caregiving responsibilities [33], and by young patients, as they are more familiar with new technologies [34]. Recently, Santesteban-Echarri et al. [35] reported advantages to videoconferencing in psychosis populations, such as higher attendance rates and patient satisfaction. In fact, during the COVID-19 pandemic, a high number of clinicians delivered MCT remotely [36]. 

Adapting MCT to include mothers with psychosis and their adolescent children (MCT-F) in an online group setting can improve cognitive awareness and the mothers’ symptoms and simultaneously enhance the family’s relationship and children’s knowledge of their mother’s disease. Studies suggest that preventive interventions for the children of mothers with psychosis should focus on providing explanations about their parent’s illness and improving cognition, social support, and cohesiveness with the local community [4,37]. By recognizing the parental role’s significance in the recovery process, suitable interventions like MCT-F also have the potential to help prevent custody loss [38], as well as mitigate the children’s risk of developing internalizing and externalizing problems [13].

Considering that mothers with schizophrenia need extensive support for themselves, their children, and their family unit as early as possible, the present study aims to adapt MCT to be delivered to mothers and their adolescent children in an online group setting with other peers. We applied the ADAP-ITT framework, which has proven to be effective for that purpose [39]. It has previously been utilized when adapting other psychological interventions and in mental health populations [40,41,42]. ADAPT-ITT was also selected as it allows for the involvement of patients, relatives, adolescents, and experts. This qualitative study also explored not only the significance of a first-person perspective but also of incorporating service providers into the design of the intervention, who could potentially deliver it. This aids in increasing adherence, considering the difficulties associated with people with psychosis and their families and the challenges in recruiting and retaining participants [43]. 

Building upon successful adaptations of psychological group programs in family settings [44], the present study aims to describe the adaptation process of MCT for mothers with psychosis and their adolescent children and demonstrate the feasibility and potential benefits of this first adaptation.

## 2. Materials and Methods

### 2.1. Design 

The protocol of this project was approved by the Ethics Committee of Sant Joan de Déu (reference: PIC-84-22). Participants were given an information sheet and all of them signed an informed consent file for participation in this study. The first six steps of the ADAPT-ITT method were conducted between September 2022 and June 2023 to adapt MCT to an intervention aimed at mothers with psychosis and their adolescent children (see Table 1 for an overview of the intervention development phases). 

### 2.2. Participants

Participants were recruited between July 2022 and February 2023, with a total of 29 enrolled in the study. Convenience and non-probabilistic sampling methods (purposive sampling) were used. The sample was comprised of adult females with a primary diagnosis of a psychotic spectrum disorder (*n* = 3), patients’ relatives (*n* = 2), adolescents aged between 12 and 17 years old (*n* = 3), and topical experts in psychosis (*n* = 21). Seven experts dropped out before completing Step 5 due to a lack of time. The procedure section indicates the phase in which each group of participants was involved.

Inclusion criteria for adult females with a primary diagnosis of a psychotic spectrum disorder were as follows: (1) to have a diagnosis of schizophrenia, unspecified psychotic disorder, delusional disorder, schizoaffective disorder, brief psychotic disorder, or schizophreniform disorder (according to DSM-5 criteria) and (2) psychopathological stability. 

Inclusion criteria for patients’ relatives were as follows: (1) be a relative to a person with a primary diagnosis of a psychotic spectrum disorder enrolment and (2) have lived with them for at least 10 years.

No other criteria were required for the adolescent group.

Finally, to be eligible, clinicians and researchers were required to provide at least 5 years of experience in psychosis, gender, and psychological interventions in adulthood and/or adolescence.

### 2.3. Measures 

MCT-F feedback and semi-structured interview. In the process of obtaining feedback on the MCT-version 1, a semi-structured interview approach was employed. During the presentation of the content of the MCT-F-v1, a semi-structured interview was used to elicit information from patients, patients’ relatives, and adolescents about their thoughts and opinions on the material. Each participant underwent a single interview with one of the three interviewers and authors (S.O., R.C., A.A., and V.E.). All interviewers, psychologists with a master’s degree, had received training for this purpose. The semi-structured interviews lasted approximately 60–90 min, involving a predetermined set of questions developed prior to the interviews but which were expanded to include additional topics that arose over the course of the study. Interviewers used open-ended question interviewing techniques (e.g., can you tell me more? What is your opinion about these examples?) to elicit further discussion and were encouraged to utilize interview questions flexibly to maintain a conversational atmosphere.

Feasibility and acceptability MCT-F questionnaire. All participants were also asked about the perceived value and usefulness of the intervention and the delivery format through a brief self-reported questionnaire divided into two parts. The first part consisted of 8 generic items and 2 specific items for each module on an 11-point Likert scale from 0 (“not at all”) to 10 (“completely”). The second part consisted of 8 short open-ended questions (e.g., What did you like about MCT-F? What did you dislike about MCT-F? What change do you suggest that should be made to MCT-F?). Although item content was generally consistent across all groups, subtle variations were introduced between the versions tailored for patients, patients’ relatives, and adolescents, as well as those for professionals. This intentional divergence aimed to capture diverse perspectives, seeking feedback both as potential participants and from professionals.

### 2.4. Procedure

#### 2.4.1. Step 1: Assessment 

The research team first approached the subject by reviewing existing literature on different topics and incorporating their considerable clinical and research experience in the field. The topics included: psychosis and motherhood (prevalence, main difficulties, and needs); the impact of parents’ mental health on their children; mental health professionals’ perception of working with parents with psychosis and their relatives; different psychological interventions, both for the so-called at-risk mental states and severe mental disorders; and the advantages and disadvantages of current interventions’ format and content. The literature review strategy was informed by a recent systematic review focused on preventive psychological interventions for children of parents with mental disorders [21] realized by the investigator principal of the research team. The keywords employed to conduct the review were tailored to align with the topic outlined in this step.

#### 2.4.2. Step 2: Decision 

In this step, consensus groups were composed of clinicians and expert researchers in psychosis; gender; evaluating psychological interventions’ efficacy and acceptability in psychosis in both adult and adolescent samples; and planning different community-focused interventions. The groups discussed the available information from Step 1 and identified the most appropriate type of psychotherapy. In addition, they decided the main themes that should be covered and which components would be included in (or excluded from) the original chosen intervention. After the work carried out by the consensus groups, the coordinating team (CT) unified the obtained information under a more homogeneous criterion and developed materials based on the topical experts’ proposals. 

#### 2.4.3. Step 3: Administration

The CT showed the initial version of the adapted intervention to the different stakeholders. Using a non-probabilistic sampling method (purposive sampling), adult females with a primary diagnosis of a psychotic spectrum disorder were recruited along with their relatives and, finally, general population adolescents aged between 12 and 17 years old. 

Researchers used semi-structured interviews to elicit information from participants about their thoughts on the material. Participants were also asked about the perceived value and usefulness of the intervention and the delivery format. The stakeholders’ appraisal was also collected through a brief self-reported questionnaire. The first part consisted of 8 generic and 2 specific items for each module on an 11-point Likert scale from 0 (“not at all”) to 10 (“completely”). The second part consisted of 8 short open-ended questions. 

#### 2.4.4. Step 4: Production 

Once feedback from the stakeholders was gathered, the CT applied it and made further modifications. In case of significant disagreements in the collected feedback, the existing literature and expert opinion were used to assess the advantages and disadvantages of each option.

#### 2.4.5. Step 5: Topical Experts 

An adapted intervention (version 3) was presented to topical experts from the consensus groups, who were then asked to assess the feasibility and readiness of the intervention through a brief 20-item questionnaire presented on an 11-point Likert scale (0 “not at all” to 10 “completely”) and 8 short open-ended questions. 

#### 2.4.6. Step 6: Integration 

Finally, the CT integrated all the feedback received from the previous steps into a final intervention version.

#### 2.4.7. Steps 7 and 8: Training and Testing 

At this point in time, the intervention is ready for staff training, including recruiters, assessment management, and therapists, and testing version 3 as part of a pilot randomized controlled study [NCT05358457] that is currently underway. 

#### 2.4.8. Data Analysis

In order to explore which main themes should be covered by the adapted intervention and to assess its feasibility, acceptability, and implementation readiness, both qualitative and quantitative methods were employed. 

In the first and second steps, we performed different thematic analyses of the information obtained through researching the literature and feedback from the consensus groups to identify what contents should be included in the intervention, as well as the emerging information on its format. In Step 1, the thematic analysis was undertaken by an extraction of themes emerging from the examined publications, together with a summary to illustrate each theme. In Step 2, a thematic analysis was used to analyze consensus group data and to develop more implicit themes from the data to either adapt or create new content or modules for the adapted intervention. Thematic analysis was conducted by the coordination team. The first phase of this analysis included the re-reading of the consensus group notes, as each member of the coordinating team had been assigned to a consensus group and participated in all the meetings held in this phase. Themes represented the experiences of multiple participants. The research team meetings allowed us to discuss thematic development and discrepancies, as well as to reach a consensus on final themes. Once the themes had been agreed upon, they were then reviewed by an external reviewer to produce the first version of the MCT-F.

In the third, fourth, and fifth steps, we analyzed the quantitative (using descriptive statistics) and qualitative (by coding responses to open-ended questions) feedback from the different stakeholders’ interviews, questionnaires, and short open-ended questions, respectively. 

Different types of triangulations were implemented to ensure the validity and transferability of the adaptation process. Data triangulation was employed in Steps 2, 3, and 5 (feedback was collected from different people and at different times). Moreover, investigator triangulation was ensured by involving several investigators throughout the whole adaptation process. Finally, methodological triangulation was carried out by using literature research by experts in Step 1, multiple consensus groups in Step 2, and qualitative and quantitative feedback analysis in Steps 3 and 5.

## 3. Results 

### 3.1. Step 1: Assessment of Needs and Different Intervention Options

The review was conducted between January 2022 and June 2022. The search strategy used keywords relating to “mother with psychosis”, “adolescence”, and “psychological intervention”. Some specific terms related to the main keywords were “schizophrenia”, “psychotic”, “psychosis”, “severe mental illness”, “mental disorder”, “child of impaired parents/psychology”, “mother”, “maternal”, “parenting intervention”, “program”, “therapy”, and “psychoeducation”. The findings highlight the impact of psychosis on parental roles, in which mothers with psychosis are more prevalent than fathers, the former of whom express significantly more risk factor needs in taking care of their children. The research explored how psychotic diagnoses affect mother–child relationships to understand their impact. Studies emphasized that psychosis indeed predicts deficits in social cognitive abilities, which affect the ability to understand their children’s mental states and, consequently, empathize and support them adequately. During adolescence, these problems in care could be aggravated, as it is a crucial stage in the development of the sense of self and metacognitive strategies. A further aim was to establish evidence on the efficacy of interventions tailored to mothers with psychosis and their adolescent children. On the one hand, this type of intervention is scarce; on the other hand, the interventions available for parents with severe mental illness are usually addressed to increase psychoeducation regarding the illness or improve parental skills; however, none aim to intervene by improving errors in cognitive biases or social cognition. Research also points out that females with schizophrenia may respond better to psychological interventions, not only in terms of improvements but also in compliance and motivation. Moreover, better cognitive functioning, a stimulating family environment, and social support were found to be protective factors against psychotic experiences among children of mothers with psychosis. Thus, the thematic analysis revealed four principal themes across the published findings: prevalence and risk factors in motherhood with psychosis, impact on social cognitive abilities, challenges during adolescence, and scarcity of tailored interventions.

In sum, the researchers concluded that the needs of mothers with psychosis and their adolescent children remain largely uncovered and that interventions for mental disorders need to target other dimensions than those currently targeted as early as possible. As a result of this step and the research group’s substantial experience in MCT in people with psychosis, it was suggested that MCT could target the current unmet needs detected in this review. 

### 3.2. Step 2: Decision 

Based on the findings from Step 1, topical experts decided to select MCT and develop an adaptation to include mothers with psychosis and their adolescent children (MCT-F) based on the core elements of this evidenced-based training. The main themes emerging from the thematic analysis based on consensus groups are reflected below. Topical experts agreed on the desirability of including an introductory module oriented to achieve the following objectives: creating a comfortable and secure environment, clarifying the objectives and structure of the training, exploring adolescents’ prior knowledge of psychosis, describing the main symptoms of psychosis, and, finally, ensuring that participants are aware of the individual, familiar, and social benefits of MCT-F. Similarly, there was consensus on adding more visual materials and reducing the amount of content in the original MCT modules. Topical experts also coincided in including current and culturally adapted examples that reflect specific situations or concerns and relevant stressors in our population (i.e., mothers with psychosis, adolescents, and mother–child relationships). Regarding delivery, the different groups pointed out the relevance of considering the advantages of MCT when applied online. This delivery would facilitate the collection of a large enough sample to run the RCT, since this specific population may not be very prevalent or easy to recruit. Finally, in many groups, discussions reflected the debate between the costs and benefits of using the term “psychosis” and addressing symptoms such as “hearing voices”. 

Discrepancies in adaptations between topical expert consensus groups were resolved by discussing it within the CT. 

After an agreement was reached, the CT integrated all proposals, producing version 1 of the intervention. The themes extracted from the thematic analysis allowed an outline of the specific domains for adaptation (see Table 2). The largest number of changes involved the language and metaphors used and content domains, and the least changed areas were concepts and goals. Changes were mainly adaptations made to optimize adherence and acceptability. MCT content was not changed beyond age-appropriate adaptations. Goals, strategies, and techniques were conserved, with edits made only to examples. The key examples of adaptations under each domain are described below and in Table 2.

### 3.3. Step 3: Administration 

Table 3 provides data on the patients’, relatives’, and adolescents’ subjective appraisal of MCT-F. Two themes emerged after participants were shown the first version of MCT-F. The first theme referred to the acceptability of the intervention content while the second theme referred to recommendations for MCT-F delivery. 

Regarding the value of the intervention, the three kinds of participants thought the program content was sufficiently comprehensive (M = 8.33; DT = 0.82) and “enough” to address the goals. They also agreed that the treatment had been helpful to them *(“I value the training positively as my family might have needed it many times and it could have been useful at key moments”).* Moreover, they did not want any of the program content removed, remarking that all the modules were potentially beneficial. Patients generally evaluated individual modules positively. The mean ratings for usefulness and enjoyment with the modules were high, ranging from 8.5 to 9.33 and from 7 to 8.67, respectively. Table 3 displays descriptive statistics for each MCT-F module. 

Most participants highlighted the relevance of included examples with which they felt a strong sense of identification because they represent familiar situations and concerns *(“I feel very close to the examples of relapse and accusation of others”; “It’s important to freely discuss drug use because it is a very common vice and judgments in patients and families with psychosis”; “I also think many times that a colleague is going to insult me or something or that I’m being followed and then this never happens”).* Mothers, especially, identified with examples regarding concern and guilt about how psychotic symptoms affect their parenting or their children *(“when I’m feeling bad I don’t feel like doing anything…Then, my daughter insists a lot on me getting dressed up… She overwhelms me”, “My biggest fear was losing my daughter”)*. In addition, improvements in the knowledge of the disease or self-knowledge and, therefore, increased capacity to empathize with others and with themselves were perceived as major benefits after reviewing the training. 

*“I was able to/could understand that everything is part of a perception and comprehend that not everything is my fault, or the fault of the other family member. It really reduced my guilt level and helped me to empathize, to understand, to raise awareness (…) I can work towards a better outcome in the relationship.”*

Nevertheless, most of them recommend refining drawings because some examples, especially in modules 4 and 5, were difficult to understand or visualize. Some adolescent participants also recommended reducing some text or explaining it in an easier way. 

Finally, participants also made recommendations for program delivery. When asked about the duration of the training, participants generally thought it was acceptable. In addition, most of them considered that the face-to-face format had the advantage of feeling closer and belonging to the group. This was especially true for the patients’ relatives (see Table 3). On the other hand, all of them recognized that the online format allows for more flexibility and intimacy, which encourages participation. There was also a high consensus on the group format. All participants agreed that sharing with others reduced stigma and loneliness. 

A patient did, however, show concern about being involved in the program with her adolescent children. This may explain the lower scores on the usefulness of this training (see Table 3). On the one hand, she felt that *“children should be kept out of the disease”* because she was afraid of conveying fear and worry or making them unhappy. On the other hand, she positively appreciated that other people with psychosis discuss this topic openly with their children. In addition, a patient’s relative also reported that he may feel self-conscious when talking about his concerns or experiences with the disease in front of the family member for fear of making him feel guilty. 

### 3.4. Step 4: Production 

Based on participants’ suggestions (Step 3), CT included an additional session after module 3, with mothers and children attending separately to enhance openly speaking about feelings or concerns without their relatives. This grants an opportunity to collect valuable feedback on the acceptability of the intervention’s first stage and make changes if necessary. CT also made some minor modifications to the intervention materials in terms of ensuring all modules were appropriate and relevant to the objective and context of the intervention. In addition, CT refined the intervention materials to enhance their appeal and comprehensibility. A summary with key examples of this adaptation’s decisions under each module is outlined in Table 4. Together, these modifications produced the second version of MCT-F. 

### 3.5. Step 5: Topical Experts 

Next, the CT asked consensus groups once again to review the second version of MCT-F. See Table 5 for descriptive statistics on the readiness for the MCT-F as rated by the professionals (*n* = 14). 

They agreed that the vocabulary and content could be understood by the study’s target population. In addition, they would neither remove nor add any content. A strength of the adapted training noted by professionals was the dynamism and appropriateness of the examples *(“I liked the varied and updated examples, adapted to the adolescent population and mothers with adolescent children”; “In addition to a therapeutic benefit, this program is often attractive to patients, making it easier for them to maintain their attention, which is deteriorated in some cases”).* They requested some guidance in selecting the most suitable examples since there were many included in the different modules. Furthermore, topical experts also suggested some minor revisions to enhance the appeal of the intervention materials. 

It is worth mentioning that some experts expressed concern about the adolescents’ adherence and proposed reducing the number of sessions. Nevertheless, the majority disagreed, arguing that MCT was already a brief program and that most of the studies demonstrated MCT’s efficacy with 10 sessions. 

When they were asked about any concerns about the potential implementation of the intervention as professionals in daily practice, they confirmed that the number and length of sessions seemed feasible. 

Finally, as seen in Table 5, mean ratings for the appropriateness of the modules were high and ranged from 8.43 to 9.21. 

### 3.6. Step 6: Integration 

All expert comments were used to make final adjustments to the intervention materials. CT integrated all findings into a final version of the intervention ready for staff training and initial feasibility testing. Finally, following the recommendations and needs of the experts, we are currently developing a therapist manual to guide the intervention. 

In sum, after following the six adaptation steps, MCT-F is made up of 10 modules, with an extra session after module 3 for mothers and children to attend separately. The two main novelties in the materials are found in the first and last modules (which were completely created ad hoc for this intervention), as well as in the inclusion of metaphors and examples that favor the generalization of the adolescent’s everyday life. 

## 4. Discussion 

To our knowledge, this is the first study to describe the process of adapting an evidence-based intervention (MCT) for mothers with psychosis and their adolescent children (MCT-F) in an online format using the first six steps of the ADAPT-ITT framework. This is one of the few brief interventions to address psychosis that is tailored to a family context. It is novel in this context, in that not only does it address patients’ symptoms and cognitive awareness, but also the family’s relationship. It also aims to improve the children’s functioning and understanding of their parent’s disease. As secondary objectives, MCT-F also aims to increase metacognition and social cognition and improve symptoms, protective factors, and the self-perception of stigma in family members. 

In the current study, topical experts were concerned about the needs of mothers with psychosis and the high risk of developing mental disorders in their children; therefore, the importance of involving children in their parent’s treatment and recovery was appropriate. Similar findings have been described previously [12,45,46]. This was discussed in conjunction with the need for the development of mother–child relational interventions that not only increase psychoeducation regarding illness but also improve errors in cognitive biases or social cognition. Recent evidence has indicated that it was not enough for family interventions in psychosis to solely aim to improve children’s understanding of their parent’s mental illness [47]. In addition, recent studies suggest that both patients with psychosis and their children could benefit from interventions targeting metacognition and social cognition [8,11,21].

Professionals also ensured that the content was acceptable and relevant, including concerns or relevant stressors of mother–child relationships. Previous parents’ reports expressed a desire for a larger focus on them as parents rather than on the effects of their mental health on their children [48]. Together, this information guided the development of MCT-F version 1. 

Our findings suggest that this novel online intervention is likely acceptable for mothers with psychosis and their adolescent children, which is consistent with previous research in this population [35,47]. MCT has demonstrated good levels of tolerability [49]. In addition, females seem to benefit more from MCT interventions than males in terms of overall symptoms, cognitive perception, and social cognition [32]. Adolescents’, patients’, and relatives’ feedback about the intervention was positive and helped guide further modifications to the intervention’s content. 

They also provided valuable feedback on the structure, format, and delivery of the intervention, optimizing the acceptability and feasibility of its implementation. This included some participants expressing a preference for a face-to-face-based delivery of the intervention as opposed to an online-based intervention. However, in the context of the COVID-19 pandemic, online delivery has been demonstrated as safe and cost-effective, and, importantly, attrition rates do not appear to be affected when the intervention is delivered in this format [50]. Recent findings have highlighted the advantages of online-based treatment in this population, i.e., females [33], adolescents [51,52], and people with psychosis [35]. Thus, online-delivered MCT could be a promising alternative intervention capable of reaching mothers unwilling to attend in-person therapy, as disengagement is a problem in early intervention for psychosis services [53], as well as for families in isolated areas [54]. Nevertheless, future research could compare the face-to-face delivery of MCT-F to online delivery and ascertain which delivery type leads to better outcomes. Based on participant feedback, we added a session where mother and child could attend in different groups and share experiences and fears with other peers in similar situations. The benefits of peer support interventions in psychosis are a robust finding in FEP research [21]. Suggestions were also made about how to tailor the materials to enhance their appeal and comprehensibility, as well as refine the content to approach symptoms and avoid upsetting participants or causing new concerns. Parents participating in family interventions suggest that content should be thoughtful to avoid parents feeling stigmatized or blamed [11]. 

Topical experts made similar suggestions for intervention refinements. They also requested ways of facilitating intervention delivery. As a result, we offered a training session for evaluators and therapists in MCT-F and added a therapist manual to guide module delivery and assure compliance with the treatment’s goals. This highlights the importance of including not only the target population but also the service providers who could potentially deliver it when adapting an intervention’s materials. Evidence suggests that adult mental health professionals feel inadequately trained to work with service users who are parents [55,56]. This may result from perceiving increased responsibility in balancing not only the safety and well-being of their patient (i.e., parents) but also that of their children. Consequently, professionals feel elevated levels of anxiety due to their role in ensuring the well-being of their patient’s children as well [47].

The current study has some limitations. Firstly, the thematic analysis in Step 1 was challenging due to heterogeneity, scarcity, and the low quality of the studies. This underscores the complexity of the subject and emphasizes the need for more robust and consistent research in this area. Secondly, while it would have been ideal to have a higher number of mothers with psychosis or children involved in Step 2, we had to prioritize patients’ availability to review all the documents deeply to guarantee the quality of the information. Nevertheless, participants’ suggestions for refining the structure and format of the intervention were congruent with the topical experts’ opinions. These factors enhance our confidence in the relevance of these findings. Another limitation involves data collection methods. Focus groups, rather than consensus groups, could have been conducted instead. A further limitation is that only one session with the mother and child attending separately was included in this study. Future trials may benefit from including more sessions separating participants by peer groups. 

A strength of this study is the participatory nature of the adaptation process, which involved patients, relatives, adolescents, mental health professionals, and researchers. Using a variety of methods (e.g., consensus group discussions, interviews, desk reviews), we triangulated data to inform evidence-based adaptations. Furthermore, since the main changes included in each phase are described and justified, this study helps to address the lack of detailed descriptions of adaptations from evidence-based interventions. Finally, MCT-F, being online-based, offers promise as an intervention that can overcome current mental health service access barriers without stigma. 

Due to the qualitative and exploratory nature of this study, we are aware that additional adaptations to the intervention may be required following further RCTs. Since deciding the number of sessions was a challenge in the adaptation process, future studies should examine the optimal number of MCT-F sessions to achieve a cost-effective balance. We also think other populations may potentially be eligible for MCT-F, such as co-parents or younger children. Lastly, as there appears to be a growing tendency for males with psychosis to become fathers, this demographic could especially benefit from the present adapted intervention. 

## 5. Conclusions 

We report the first online MCT adaptation for mothers with psychosis and their adolescent children. The adaptation process highlighted the importance of working using a first-person perspective to ensure that the intervention adequately addressed issues faced not only by individuals but also by professionals involved in the delivery of the intervention. Once MCT-F’s feasibility and efficacy are confirmed with RCT, it could become the new tool to help mothers experiencing psychosis and their adolescent children who are known to be at higher risk of developing a several mental disorder. 

## Figures and Tables

**Figure 1:**
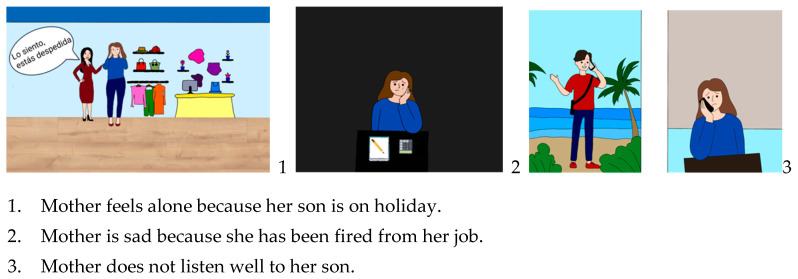
This exercise aimed to address the bias against disconformity evidence. It consists of a series of three images shown in reverse order. The sequence of images gradually reveals a complex story. For each image, participants are asked to evaluate the plausibility of the three different interpretations. At the end of each test, the correct interpretation is discovered.

**Table 1:** Applying the ADAPT-ITT model to MCT to create Family Metacognitive Training (MCT-F) for mothers with psychosis and their adolescent children.

ADAPT-ITT	Method	Changes to Intervention
Step 1: Assessment	A research team first approached the subject by reviewing existing literature on different topics and based on their clinical and research experience in the field (the research team included different experts in psychosis, gender, adulthood, adolescence, psychological interventions, and metacognition).	
Step 2: Decision	Different topical expert consensus groups discussed the findings from Step 1 and selected MCT. They also identified main themes and specified components for inclusion/exclusion from the original MCT The coordinating team developed materials (MCT-F-v1) based on the topical experts’ proposals.	MCT-F-v1
Step 3: Administration	The different stakeholders (patients, relatives, and adolescents) received the first version and gave initial feedback about the acceptance, feasibility, and attractiveness of the material.	MCT-F-v1
Step 4: Production	The feedback from Step 3 was used by the coordinating team to further modify the content and structure to produce version 2.	MCT-F-v2
Step 5: Topical Experts	Topical experts from the consensus groups were asked to evaluate the current adaptation and suggest other necessary modifications.	MCT-F-v2
Step 6: Integration	All findings and feedback were integrated to produce the final version of the adapted intervention.	MCT-F-v3
Step 7: Training	There will be different training for recruiters, facilitators, and assessors to implement the final MCT-F version.	
Step 8: Testing	A pilot randomized controlled trial comparing MCT-F with a waiting list control group is being planned.	

**Table 2:** Key adaptations to MCT for mothers with psychosis and their adolescent children (MCT-F) in Step 2.

Domain	Description	Examples of Adaptations
Context	Increase accessibility; enhance feasibility, acceptability, and compliance	Mothers and children will be able to attend sessions from the same place or separately to facilitate attendance and the intimacy of both parties. Group composition: The groups will be composed of 3–4 mothers with psychosis, their adolescent children, and two therapists. Groups will be formed based on the adolescents’ ages (aged 12 to 16 and 16 to 20) to adapt examples and vocabulary and so they feel more comfortable sharing experiences. Group size: A group of 3 to 4 mothers and adolescents is large enough in case of some participant absences cause those attending to feel exposed. It is also manageable for two facilitators.
Persons	Engaging non-mental health adolescent children of mothers with psychosis; assess the tolerability and effectiveness of the MCT-F in mothers and their adolescent children. Promoting the therapist–patient relationship	Application of MCT-F to mothers with psychosis together with their adolescent children in a peer group setting. Therapists will also provide local community references for the mother’s participation.
Goal	Clarify and extend goals; improving the children’s knowledge of the disease.	*Module 1*: Adding content to help adolescents to understand the symptoms of their mother’s disease. Linking MCT-F to healthy populations (adolescents). Describing MCT-F as a training program aiming to increase cognitive flexibility, modify metacognitive beliefs, and decrease dysfunctional coping strategies.
Language	Ensuring translation is harmonious with adolescents’ language; replacement of technical terms with colloquialisms. Ensuring Spanish translation is gender inclusive.	*Module 2:* “Attribution = to infer causes about events” is replaced by “Attribution = to give an explanation or look for a cause of a situation”; “Megalomaniac” is replaced by “thinking we are more important than others”. *Module 9:* A Spanish metaphor is included to explain the cognitive distortion of overgeneralizing: “I killed a dog, then they called me dog killer”; “Depressive attributional style” is replaced by “depressive thinking”; “Comprehensive assessment” is replaced by “take everything into account”. All modules: use both sexes in verbs, determinants, pronouns, etc.
Concept	Ensuring concepts of mentally ill health are understood.	*Module 2:* The content dedicated to addressing the symptoms of “hearing voices” includes examples like impulsion phobias which may be more familiar to adolescent children and make them easier to understand (e.g., “Thirty-nine and forty-three percent of males and females, respectively, had had the intrusive thought of jumping from a high place.”).
Method	Promoting adolescent engagement; adapting the intervention structure.	*All modules:* Worksheets were not included in this first adaptation. The number of modules from the 10-module version of MCT (eight modules and two additional modules) was reduced to 9. The self-esteem additional module is added to the self-esteem and mood module. The content of the relapse prevention worksheet is added to the module dealing with self-stigmatization.
Metaphors and content	Promoting engagement. Ensuring content is relevant and acceptable for adolescent age groups using current examples. Including examples of relevant stressors for mothers with psychosis, adolescents, and the mother–child relationship.	An introductory and psychoeducational module is added. *All modules:* Photos with young people (laughing, partying, using the cell phone, etc.) are included. *Module 3*: Jumping to conclusions in social media (e.g., “Seeing that a follower on Instagram or TikTok has uploaded the same photo or video as you”) or in the COVID-19 pandemic context; challenging false beliefs regarding cannabis consumption. *Module 6:* Next to the sentence “How can I memorize things better?” is an image of a brain exercising. *Module 8:* The picture of a screaming mouth and a frightened boy representing the delusions is deleted. *Module 9:* Added a picture of a hamster rolling on a wheel to represent rumination. Module 10. Added teens’ icons who opened up about their mental health disorders; added data on the prevalence of mental disorders in females and adolescents due to COVID-19. *Module 2:* The following example is added: Ana’s mother has had a relapse and has been admitted. Ana’s thoughts: the relapse was caused because my grades have gotten worse. Another explanation: Ana’s mother had stopped taking her medication. Moreover, she had had an argument at work and was afraid of losing her job. *Module 4:* Figure 1 shows an example of new material added to address the bias against disconformity evidence through a possible common situation in the mother–child relationship. *Module 8:* The following example is added: “Example: Judith’s daughter’s volleyball coach proposes to Judith that her daughter competes this weekend. Background: She is convinced that the coach wants to take away her role as a mother and push them apart. This weekend is Judith’s birthday. But: The coach wants the best for her daughter. Besides, she’s keeping Judith in mind”. *Module 9:* Exercise including “What do you like about your mother? What is your son/daughter good at?”; Add examples of common cognitive bias among adolescents or in the mother–child relationship (e.g., “I shouted at my son/daughter the other day so I’m a bad mother”).

**Table 3:** Feasibility and acceptability for MCT-F version 1 rated by the participants (*n* = 8).

Variable		Patients	Patients’ Relatives	Adolescents	Total
		M (SD)	M (SD)	M (SD)	M (SD)
Do you think the vocabulary of the modules is comprehensive?		8.00	7.50 (0.71)	9.00	8.33 (0.82)
Were you able to understand the explanations, exercises, and examples?		9.50 (0.71)	7.00 (0.00)	9.67 (0.58)	8.67 (1.37)
How easy would it be for you to incorporate an online program of 10 sessions a week at a frequency of 1 h in the afternoon into your routine?		7.50 (2.12)	8.00 (0.00)	9.00 (1.00)	8.17 (1.33)
Do you think the online format is appropriate for this type of training?		9.00 (1.41)	6.00 (0.00)	7.33 (2.10)	7.00 (1.60)
Would you recommend the training to others?		7.00 (2.83)	9.50 (0.71)	9.67 (0.58)	8.83 (1.94)
How much did you enjoy the training and did you find it attractive?		9.00 (1.41)	9.00 (0.00)	8.00 (2.00)	8.33 (1.37)
In general, did you find the training helpful (for you)?		7.50 (3.53)	9.00 (0.00)	9.67 (0.58)	8.67 (1.86)
How useful could this training be for the mother–child relationship in your experience?		5.00 (7.07)	9.00 (1.41)	9.33 (1.15)	7.67 (3.88)
M1	usefulness	9.00 (1.41)	8.50 (2.12)	9.50 (0.71)	8.80 (1.30)
	enjoyment	9.00 (1.41)	8.50 (2.12)	8.67 (0.58)	8.50 (1.05)
M2	usefulness	9.00 (1.41)	9.00 (1.41)	9.00 (1.00)	8.83 (0.98)
	enjoyment	8.50 (2.12)	9.00 (1.41)	9.00 (1.00)	8.67 (1.21)
M3	usefulness	8.50 (2.12)	8.50 (2,12)	9,00 (1.00)	8.50 (1.38)
	enjoyment	8.50 (2.12)	8.00 (2.83)	9.00 (1.00)	8.33 (1.63)
M4	usefulness	8.50 (2.12)	9.50 (0.71)	9.33 (0.58)	9.00 (1.09)
	enjoyment	8.00 (2.83)	9.50 (0.71)	9.00 (1.00)	8.67 (1.50)
M5	usefulness	9.00 (1.41)	9.50 (0.71)	9.00 (1.00)	9.00 (0.89)
	enjoyment	9.00 (1.41)	8.50 (2.12)	8.33 (0.58)	8.33 (1.03)
M6	usefulness	10.00 (0.00)	8.50 (2.12)	9.00 (0.58)	9.33 (1.21)
	enjoyment	10.00 (0.00)	7.50 (2.12)	9.00 (1.73)	8.67 (1.75)
M7	usefulness	8.50 (2.12)	8.50 (2.12)	9.00 (1.00)	8.50 (1.38)
	enjoyment	8.50 (2.12)	6.50 (0.71)	8.00 (1.73)	7.33 (1.37)
M8	usefulness	7.50 (3.53)	8.00 (2.83)	8.33 (1.53)	7.67 (2.06)
	enjoyment	9.00 (1.41)	6.50 (0.71)	7.00 (1.73)	7.00 (1.26)
M9	usefulness	9.50 (0.71)	9.00 (1.41)	9.33 (1.15)	9.17 (0.98)
	enjoyment	7.50 (3.53)	8.00 (1.41)	8.67 (0.58)	7.83 (1.60)
M10	usefulness	7.50 (0.71)	9.50 (0.71)	8.67 (1.53)	8.83 (1.17)
	enjoyment	8.50 (2.12)	8.00 (0.00)	7.67 (1.15)	8.17 (1.17)

*Note.* M, Module. Ratings were made on a 10-point Likert scale (0 = completely disagree and 5 = completely agree).

**Table 4:** Key adaptations to MCT for mothers with psychosis and their adolescent children (MCT-F) in Step 4 (MCT-F).

Module	Aim	Examples of Modified Text and New Activities
1	Promote acceptability and reduce possible defenses and fears of participating mothers about talking to their children about psychosis.	The description of psychosis and symptoms is moved from the start to the middle of the presentation. Some content from relapse prevention (early warning symptoms) is brought forward in this first module. Therapists use the vocabulary proposed by the group to refer to crises or disease outbreaks.
2	Approach symptoms such as hearing voices very carefully and avoid new concerns.	A photo of an aggressive mouth representing “voices” is replaced by an image of an adolescent girl covering her ears.
3	Enhance adherence and, consequently, increase its acceptability and efficacy.	Task Set 1 examples are narrowed down by selecting the most attractive ones.
4	Refine the intervention materials to enhance their appeal and comprehensibility.	The text and content of some comics are modified.
5	Refine the intervention materials to enhance their appeal and comprehensibility.	Some comics are redesigned and colored.
6	Address relevant stressors for participants.	Add situations related to academic issues (e.g., a picture of a classroom, “meeting with your child’s teacher” as an example of an event to work on).
7	Ensure content is easily understandable to participants.	Add numbering to comic book bullets.
8	Simplify the language used, reducing the amount of text and slides, and adding more visuals.	Task Set 1 examples are narrowed down by selecting the most attractive ones. “This type of decision-making can easily lead to errors, compared to a type of decision-making that involves careful consideration of all available information” is replaced by “+information, +certain, -information, + mistakes”.
9	Increase acceptability and adherence.	The content order is reversed. The first part addresses depression and the second addresses self-esteem to finish the session on a positive note.
10	Refine the intervention materials to enhance their appeal and comprehensibility.	Text is converted into bullet points. An image of a girl looking at herself in the mirror, thinking “I am not normal”, is added to the self-stigmatization slide.

**Table 5:** Expert’s perceived readiness for MCT-F version 2 (Step 5) (*n* = 14).

	M (DT)
Do you think the vocabulary of the modules is understandable for both adult and adolescent populations?	8.86 (1.03)
Are the explanations, exercises, and examples comprehensive?	9.00 (0.68)
How feasible would it be for a patient and her adolescent to incorporate an online program of 10 weekly 1-h afternoon sessions into their routine?	7.07 (1.38)
Do you think the online format is appropriate for this type of training?	7.43 (2.76)
Would you recommend the training to patients who meet the criteria?	9.36 (1.01)
In general, do you think training is helpful for women with psychosis who are mothers?	9.36 (0.74)
In general, do you think training is useful for adolescent sons and daughters of women with psychosis?	8.86 (0.95)
In general, can the training be useful for the relationship between mother and adolescent relationship?	8.86 (0.53)
As a professional, how feasible would it be for you to conduct training with this format considering frequency, duration, and number of sessions (10 weekly sessions of 1 h duration)?	8.36 (1.28)
As a professional, how comfortable would you feel doing this training?	8.64 (1.34)
Module 1	9.21 (0.89)
Module 2	8.43 (1.55)
Module 3	8.64 (1.34)
Module 4	8.64 (1.21)
Module 5	8.78 (1.05)
Module 6	9.00 (0.88)
Module 7	8.64 (1.21)
Module 8	8.71 (0.82)
Module 9	9.00 (1.11)
Module 10	9.14 (0.95)

## Data Availability

Due to confidentiality issues, access to data will only be granted on request (S.O.; susana.ochoa@sjd.es).
